# Supplementary material for: IRE1α determines ferroptosis sensitivity through regulation of glutathione synthesis
Source: Nat Commun. 2024 May 15;15:4114. doi: 10.1038/s41467-024-48330-0 (PMC11096184; doi:10.1038/s41467-024-48330-0)
Supplement: Supplementary file 1 — Supplementary Information [file 41467_2024_48330_MOESM1_ESM.pdf]

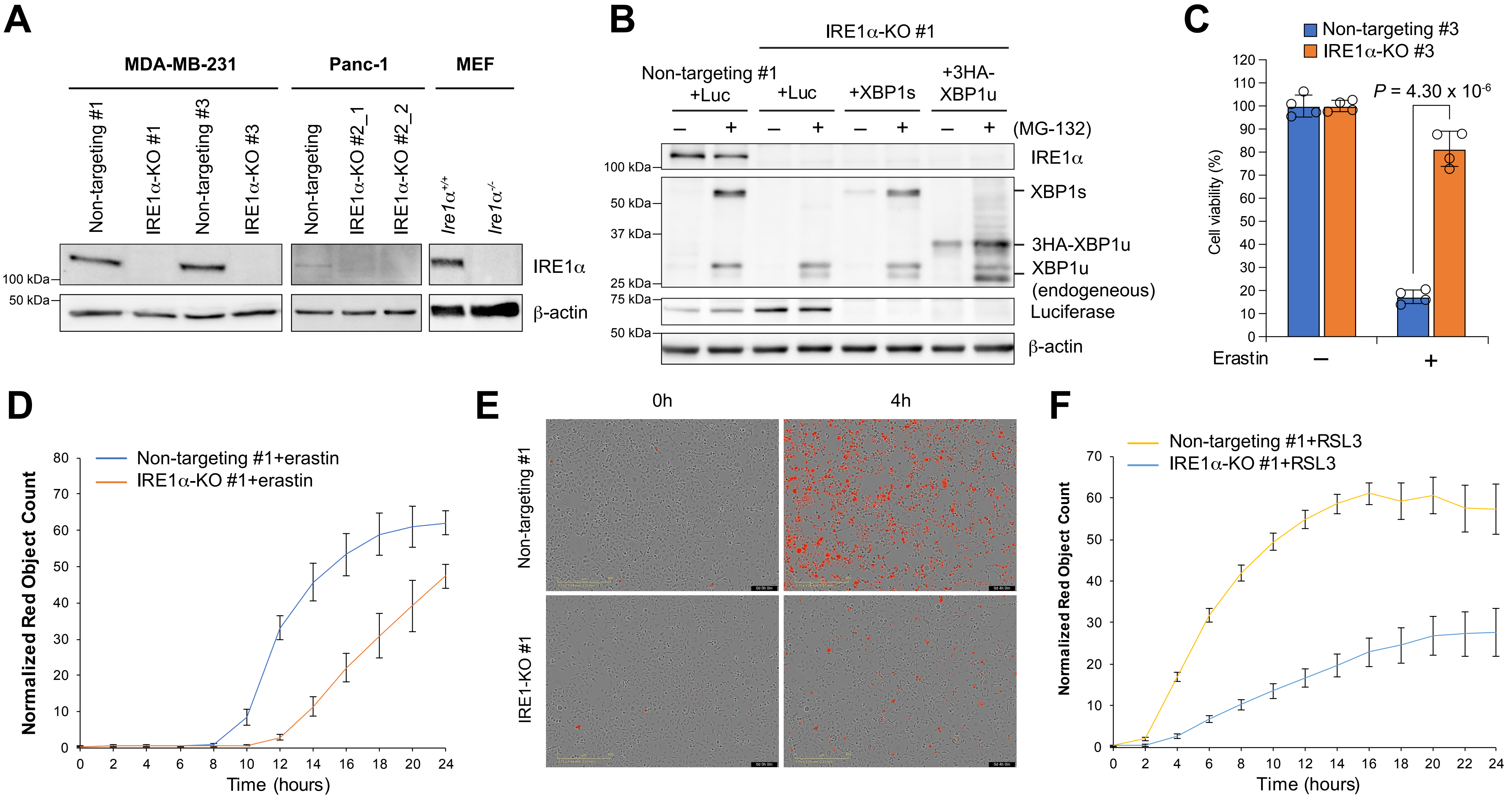

Supplementary Figure 1. IRE1α determines sensitivity to ferroptosis in additional cancer cell lines. A. Western blotting analysis of IRE1α expression in control (non-targeting) and IRE1α-null cell lines. β-actin was used as a loading control. **B**. Western blotting analysis of luciferase, IRE1α and XBP1 expression in control MDA-MB-231 cells overexpressing luciferase and IRE1α-null cells overexpressing either luciferase (Luc), XBP1s or XBP1u (with a 3HA tag). β-actin was used as a loading control. The proteasome inhibitor MG-132 (10 μM, 7 h) was used to stabilize XBP1s and XBP1u proteins. **C**. Viabilities of an additional pair of non-targeting and IRE1α-null MDA-MB-231 cells from an independent sgRNA (#3) upon treatment with 5 μM erastin. 24 hours after erastin treatment, cell viability was measured by CCK8 assay. Viability with DMSO-only treatment (erastin -) was set as 100%. **D**. Non-targeting and IRE1α-null MDA-MB-231 cells were subjected to IncuCyte Cytotox Red staining & imaging upon treatment with 10 μM erastin. The Red Object Counts normalized to cell confluency over 24 hours are plotted. **E**. Non-targeting and IRE1α-null MDA-MB-231 cells were subjected to IncuCyte Cytotox Red staining & imaging upon treatment with 1 μM RSL3. Representative phase+red fluorescence channel images from 0 and 4 hours are shown. The Red Object Counts normalized to cell confluency over 24 hours are plotted in **F**. Data are presented as mean ± s.d. in **C**, **D**, and **F**, with n = 4 in **C** and n = 36 (images) in **D** and **F**. In **C**, n indicates independent repeats. Unpaired, two-tailed Student's t tests were performed to calculate the P values for the statistical analyses. Source data are provided as a Source Data file.

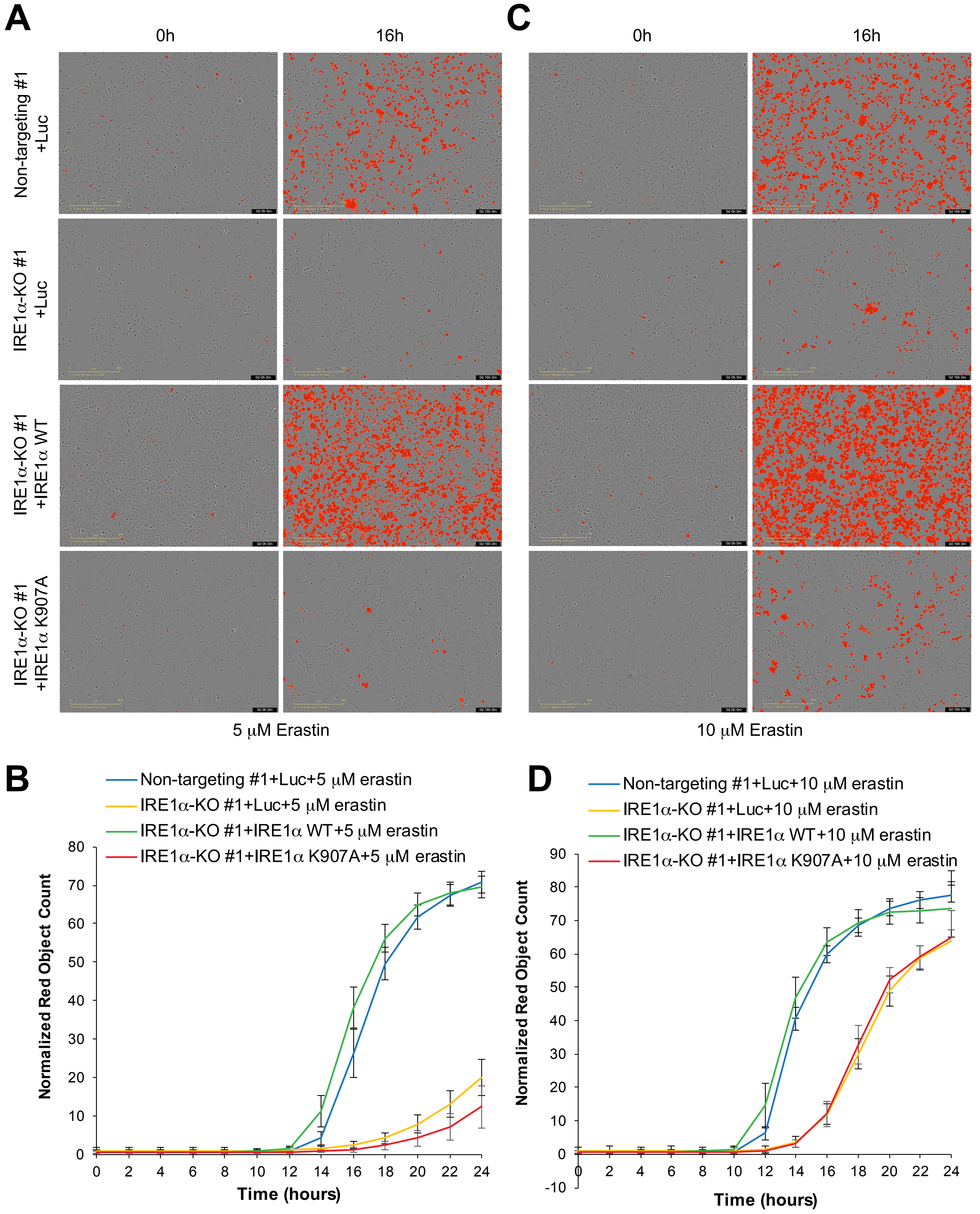

Supplementary Figure 2. The RNase activity of IRE1α determines sensitivity to ferroptosis. Non-targeting and IRE1α-null MDA-MB-231 cells reconstituted with luciferase control (Luc), wild-type IRE1α (WT) or RNase-dead K907A mutant IRE1α were subjected to IncuCyte Cytotox Red staining & imaging upon treatment with erastin. Representative phase+red fluorescence channel images from 0 and 16 hours are shown (**A** and **C**, 5 and 10 μM erastin, respectively). The corresponding Red Object Counts normalized to cell confluency over 24 hours are plotted (**B** and **D**). Data are presented as mean ± s.d. in **B** and **D** and the quantification is based on *n* = 36 images in each group. Source data are provided as a Source Data file.

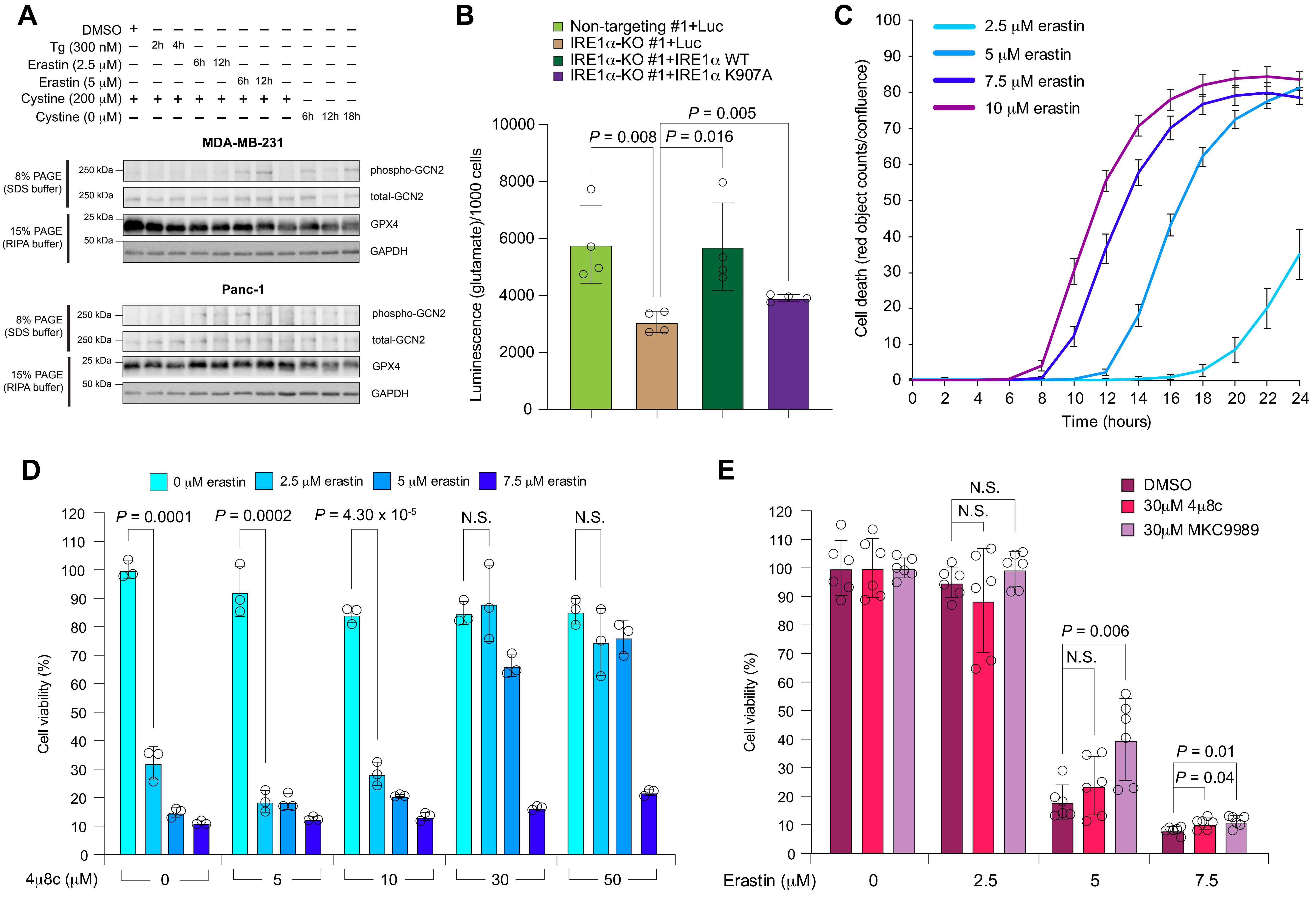

Supplementary Figure 3. A. MDA-MB-231 (top) and Panc-1 (bottom) cells were treated with thapsigargin (Tg), erastin, or cystine starvation with the indicated concentration and duration, then the expression of phospho-GCN2, total GCN2 and GPX4 were analyzed with Western blotting with the conditions shown on the left side of the blots. Total GCN2 and GAPDH were used as loading controls. **B**. Measurement of cellular glutamate levels in MDA-MB-231 cells grouped into control (non-targeting) and IRE1α-null, as well as non-targeting overexpressing luciferase (Luc), IRE1α-null overexpressing Luc, wild-type IRE1α (WT) or the K907A mutant IRE1α. The luminescence readings were normalized to cell numbers (/1000 cells). **C**. MDA-MB-231 cells were incubated with increasing concentrations of erastin and subjected to IncuCyte Cytotox Red staining & imaging. The corresponding Red Object Counts normalized to cell confluency over 24 hours are plotted. **D**. MDA-MB-231 cells treated with increasing concentrations of erastin combined with increasing concentrations of the IRE1α inhibitor 4μ8c were subjected to viability measurement with CCK8 assay after 24 hours. 0 μM erastin with 0 μM 4μ8c (DMSO+DMSO) was set as 100% in viability. **E**. IRE1α-null MDA-MB-231 cells treated with increasing concentrations of erastin combined with different IRE1α inhibitors were subjected to viability measurement with CCK8 assay after 24 hours. 0 μM erastin (DMSO only) was set as 100% in viability. Data are presented as mean ± s.d. in **B** (*n* = 4), **C** (*n* = 36 images), **D** (*n* = 3) and **E** (*n* = 6). In **B**, **D** and **E**, *n* indicates independent repeats. Unpaired, two-tailed Student's *t* tests were performed to calculate the *P* values for all the statistical analyses. N.S.: not significant. Source data are provided as a Source Data file.

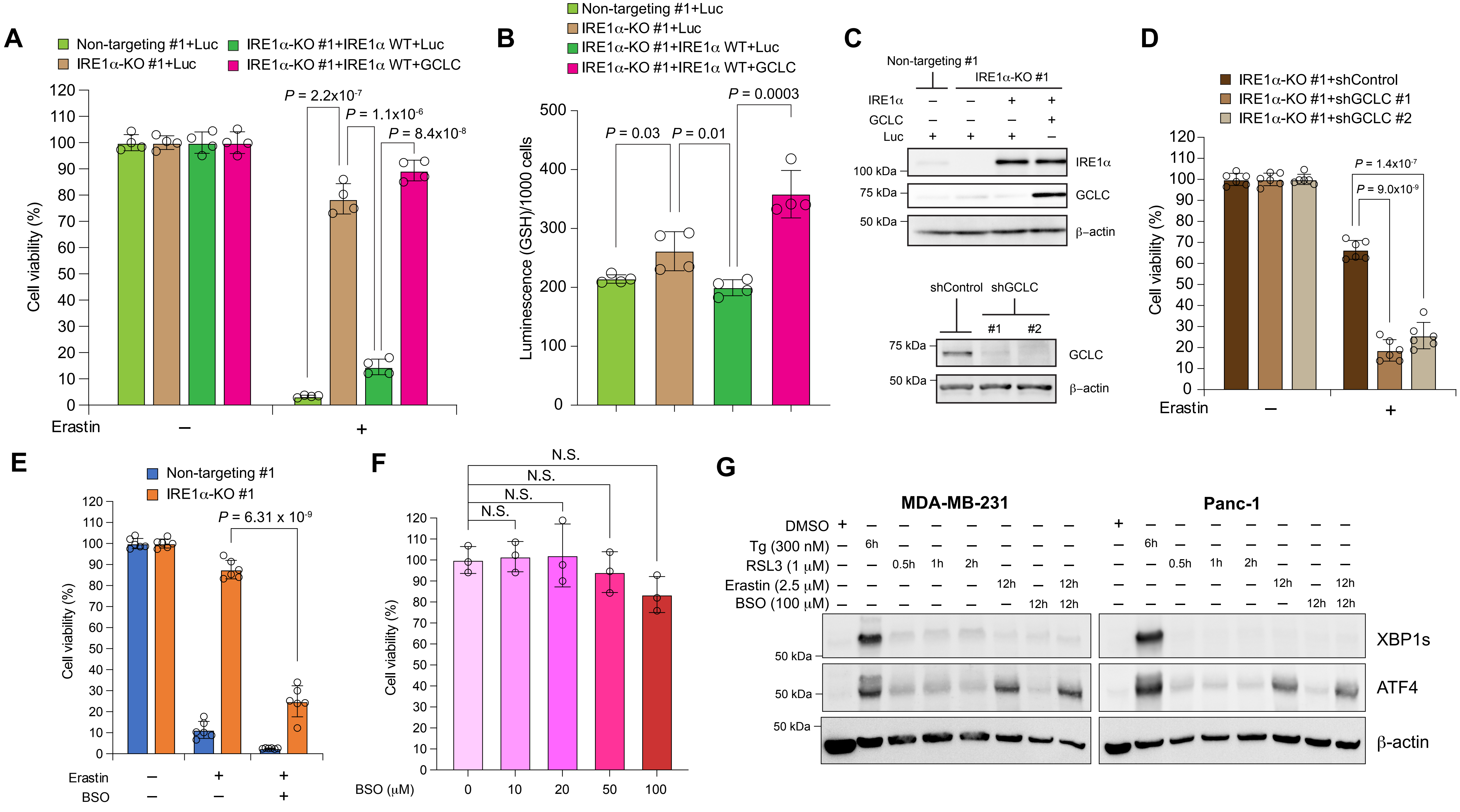

Supplementary Figure 4. IRE1α regulates ferroptosis sensitivity through GCLC. A. The viabilities of control (non-targeting) and IRE1α-null MDA-MB-231 cells overexpressing luciferase (Luc), IRE1α-null MDA-MB-231 cells overexpressing wild-type (WT) IRE1α, and IRE1α-null MDA-MB-231 cells overexpressing both IRE1α WT and GCLC after 2.5 μM erastin treatment for 24 hours measured by CCK8 assay. Viability values were normalized to treatments with 0 μM erastin (DMSO only) which were set to be 100% viable. Luciferase was used as an overexpression control. **B**. Measurement of cellular glutathione (GSH) levels in the same groups in **A** after 2.5 μM erastin treatment for 12 hours. The luminescence readings are normalized to cell numbers (/1000 cells). **C**. Top panel: Western blotting analysis of IRE1α and GCLC expression in the cell lines in **A** and **B**. β-actin was used as a loading control. Bottom panel: Western blotting analysis of GCLC expression in the cell lines in **D**. β-actin was used as a loading control. **D**. The viabilities of IRE1α-null MDA-MB-231 cells with control (shControl) or GCLC knockdown (shGCLC) after 5 μM erastin treatment for 24 hours measured by CCK8 assay. Viability values were normalized to treatments with 0 μM erastin (DMSO only) which were set to be 100% viable. **E**. The viabilities of control (non-targeting) and IRE1α-null MDA-MB-231 cells after 24 hours' treatment with 2.5 μM erastin with and without co-treatment with 100 μM glutamate cysteine ligase (GCL) inhibitor buthionine sulfoximine (BSO) measured by CCK8 assay. Viability values were normalized to treatments with 0 μM erastin and BSO (DMSO only) which were set to be 100% viable. **F**. The viabilities of MDA-MB-231 cells treated with increasing concentrations of BSO for 24 hours measured by CCK8 assay. Viability values were normalized to the treatment with 0 μM BSO (DMSO only) which was set to be 100% viable. **G**. MDA-MB-231 (left panel) and Panc-1 (right panel) cells were treated with thapsigargin (Tg), RSL3, erastin or BSO with the indicated concentration and duration, then the expression of XBP1s and ATF4 were analyzed with Western blotting. β-actin was used as a loading control. Data are presented as mean ± s.d. in all histograms, with $n = 4$ in **A** and **B**, $n = 6$ in **D** and **E**, and $n = 3$ in **F**. n indicates independent repeats. Unpaired, two-tailed Student's t tests were performed to calculate the P values for all the statistical analyses. Source data are provided as a Source Data file.

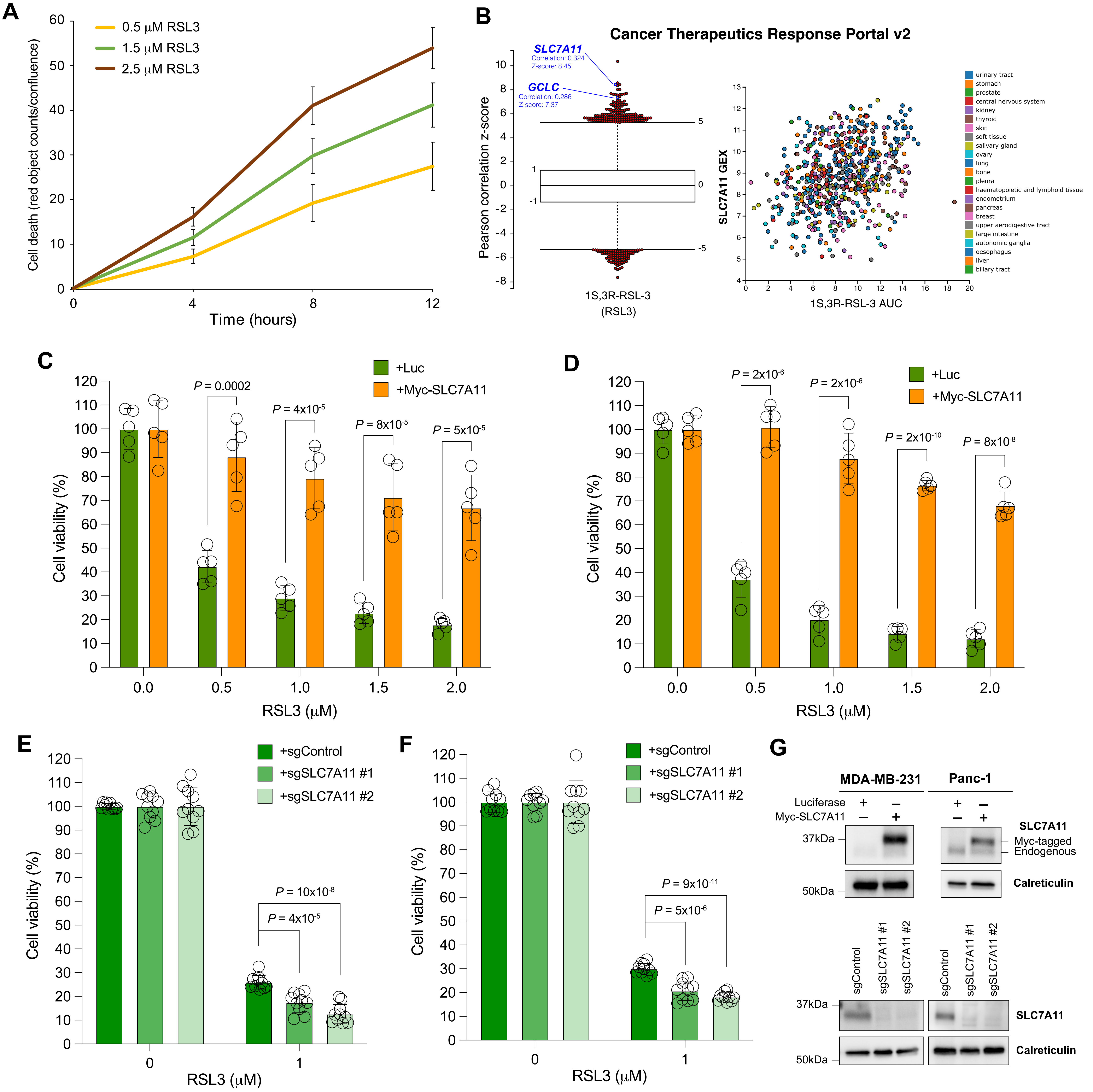

Supplementary Figure 5. A. MDA-MB-231 cells were incubated with increasing concentrations of RSL3 and subjected to IncuCyte Cytotox Red staining & imaging. The corresponding Red Object Counts normalized to cell confluency over 12 hours are plotted, as there was no further increase after 12 hours. **B**. In the Cancer Therapeutic Response Portal database (CTRP, v2), *SLC7A11* and *GCLC* are within the top genes which show resistance correlation with 1S,3R-RSL-3 (RSL3). CTRP uses combination of small-molecule sensitivity data, expressed as areas under concentration-response curves (AUCs), with genome-wide basal gene-expression measurements, expressed as $\log_2$ robust-multi-array-average values, across cancer cell lines (CCLs) to calculate z-scored Pearson expression–sensitivity correlation coefficients. Higher AUC values mean more resistance to RSL3. MDA-MB-231 (**C**) and Panc-1 (**D**) cells overexpressing control luciferase (Luc) or SLC7A11 were treated with increasing concentration of RSL3 then subjected to viability measurement with CCK8 assay after 24 hours. 0 μM RSL3 was set as 100% in viability. MDA-MB-231 (**E**) and Panc-1 (**F**) cells transduced with Cas9/sgRNA-expressing lentivirus were treated with 0 μM (DMSO) or 1 μM RSL3 then subjected to viability measurement with CCK8 assay after 24 hours. DMSO-treated cells were set as 100% in viability. Control (sgControl) and two independent sgRNAs targeting SLC7A11 (sgSLC7A11 #1 and #2) were used. **G**. Western blotting analysis to confirm SLC7A11 overexpression (top panel) and knockdown (bottom panel) in cells in **C-D** and **E-F**. Calreticulin was used as a loading control for membrane fraction. Data are presented as mean ± s.d. in **A** (n = 36 images), **C** and **D** (n = 5), **E** and **F** (n = 10). In **C-F** n indicates independent repeats. Unpaired, two-tailed Student's t tests were performed to calculate the P values for all the statistical analyses. Source data are provided as a Source Data file.

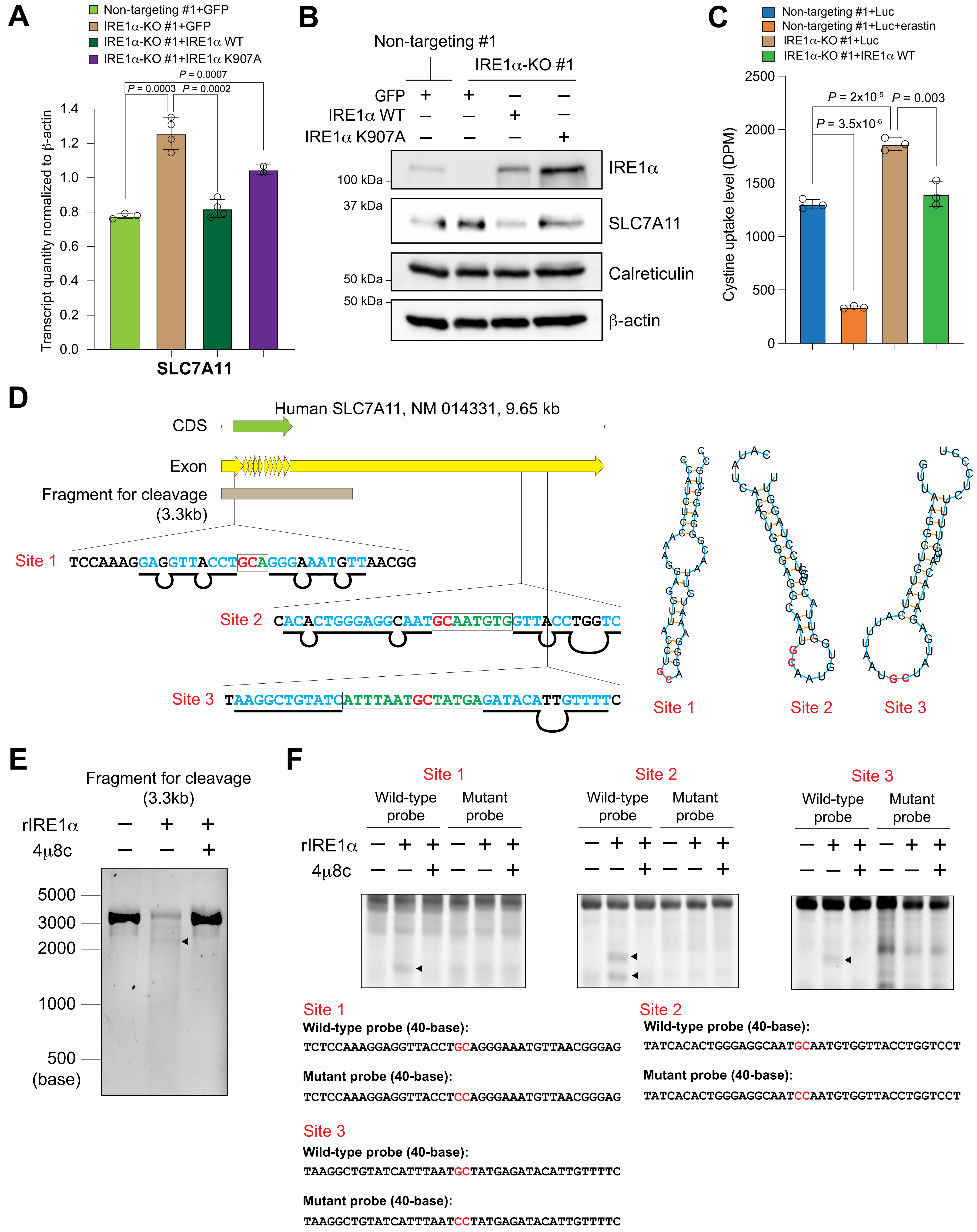

Supplementary Figure 6. SLC7A11 is a target of the RIDD activity of IRE1α. A. Quantitative real-time PCR analysis of SLC7A11 mRNA expression levels in control (non-targeting) and IRE1α-null MDA-MB-231 cells overexpressing GFP, and IRE1α-null MDA-MB-231 cells overexpressing wild-type (WT) or K907A mutant IRE1α. GFP was used as an overexpression control. β-actin was used as a normalization control. **B**. Western blotting analysis of IRE1α and SLC7A11 protein expression levels in the membrane fraction of cell lines in **A**. Calreticulin was used as a membrane fraction loading control and β-actin as a whole-cell loading control. **C**. Cystine uptake levels of control (non-targeting) and IRE1α-null MDA-MB-231 cells overexpressing luciferase (Luc), and IRE1α-null MDA-MB-231 cells overexpressing wild-type IRE1α (WT). Luc was used as an overexpression control. The control+Luc cells were also treated with 1 μM erastin to inhibit cystine uptake as a validation of the assay. **D**. Left panel: schematic view of human SLC7A11 transcript with coding sequence (CDS) region shown on the top (green) and exon boundaries in the middle (yellow). A 3.3 kb fragment (light brown) from the Mammalian Gene Collection (MGC) cDNA Clone 4562994 that was used for the IRE1α cleavage assay is shown in light brown. The sequences flanking the identified IRE1α cleavage sites are shown with the GC cleavage site marked in red, sequence in the loop part in green and sequence in the stem part in light blue. The pairing bases in the stem part are marked with black underlines and the unpaired bases are marked with black loops. Right panel: the predicted secondary structures of the mRNA sequences flanking the identified SLC7A11 IRE1α cleavage sites. The G-C cleavage sites are colored in red. **E**. *In vitro* RNA cleavage assay using the 3.3 kb human GCLC mRNA fragment, incubated in the presence or absence of recombinant cytosolic portion of IRE1α (rIRE1α). Experiments were performed in the presence or absence of 30 μM IRE1α inhibitor 4μ8c. The cleaved band is marked with a black arrowhead. **F**. *In vitro* RNA cleavage assay using wild-type and cleavage site-mutated (GC to CC) 40-base RNA oligos from the identified IRE1α cleavage sites, incubated in the presence or absence of rIRE1α and 4μ8c. The cleaved bands are marked with black arrowheads. The bands in **E** are visualized on an agarose gel and those in **F** are visualized on PAGE gels. Data are presented as mean ± s.d. in **A** (n = 4) and **C** with (n = 3). n indicates independent repeats. Unpaired, two-tailed Student's t tests were performed to calculate the P values for all the statistical analyses. Source data are provided as a Source Data file.

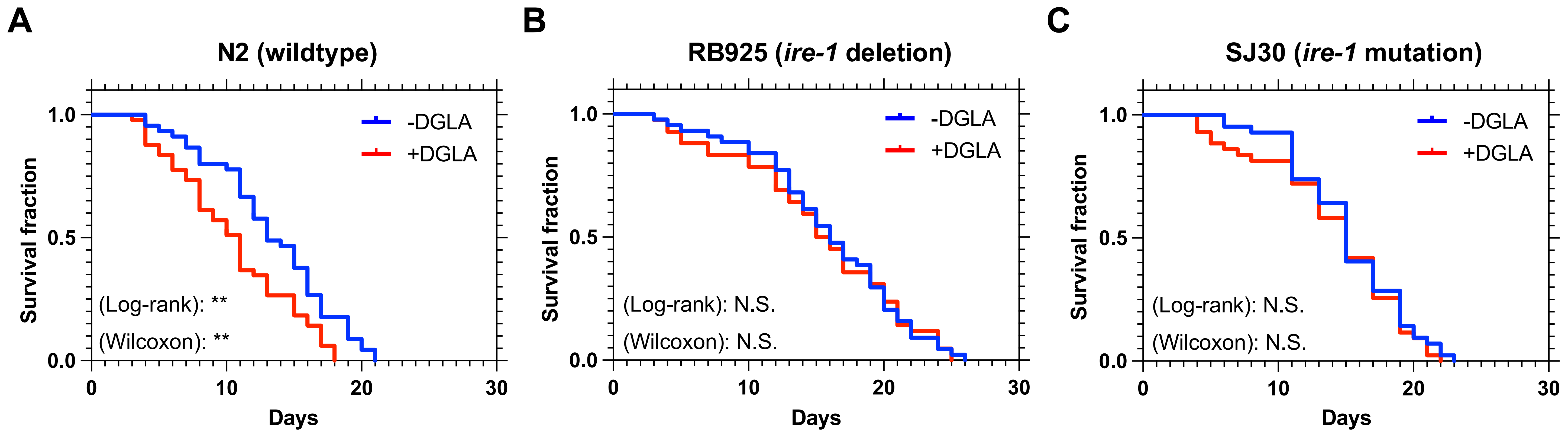

Supplementary Figure 7. IRE1α regulates ferroptosis sensitivity in an independent cohort of *C. elegans*. Kaplan-Meier survival curve of wild-type (N2, **A**), *ire-1* deleted (RB925, **B**) and *ire-1* mutated (SJ30, **C**) *C. elegans* with or without dietary ingestion of the polyunsaturated fatty acid dihomogamma-linolenic acid (DGLA). Both Log-rank (Mantel-Cox) and Gehan-Breslow-Wilcoxon tests were used to calculate the *P* values of the survival analysis. In **A**, *n* = 45 (N2-DGLA) and *n* = 49 (N2+DGLA). In **B**, *n* = 44 (RB925-DGLA) and *n* = 42 (RB925+DGLA). In **C**, *n* = 42 (SJ30-DGLA) and *n* = 43 (SJ30+DGLA). ** $P \leq 0.01$. N.S.: not significant. Median survival: N2: 13 (-DGLA) and 11 (+DGLA); RB925: 16 (-DGLA) and 15 (+DGLA); SJ30: 15 (-DGLA) and 15 (+DGLA). Source data are provided as a Source Data file.

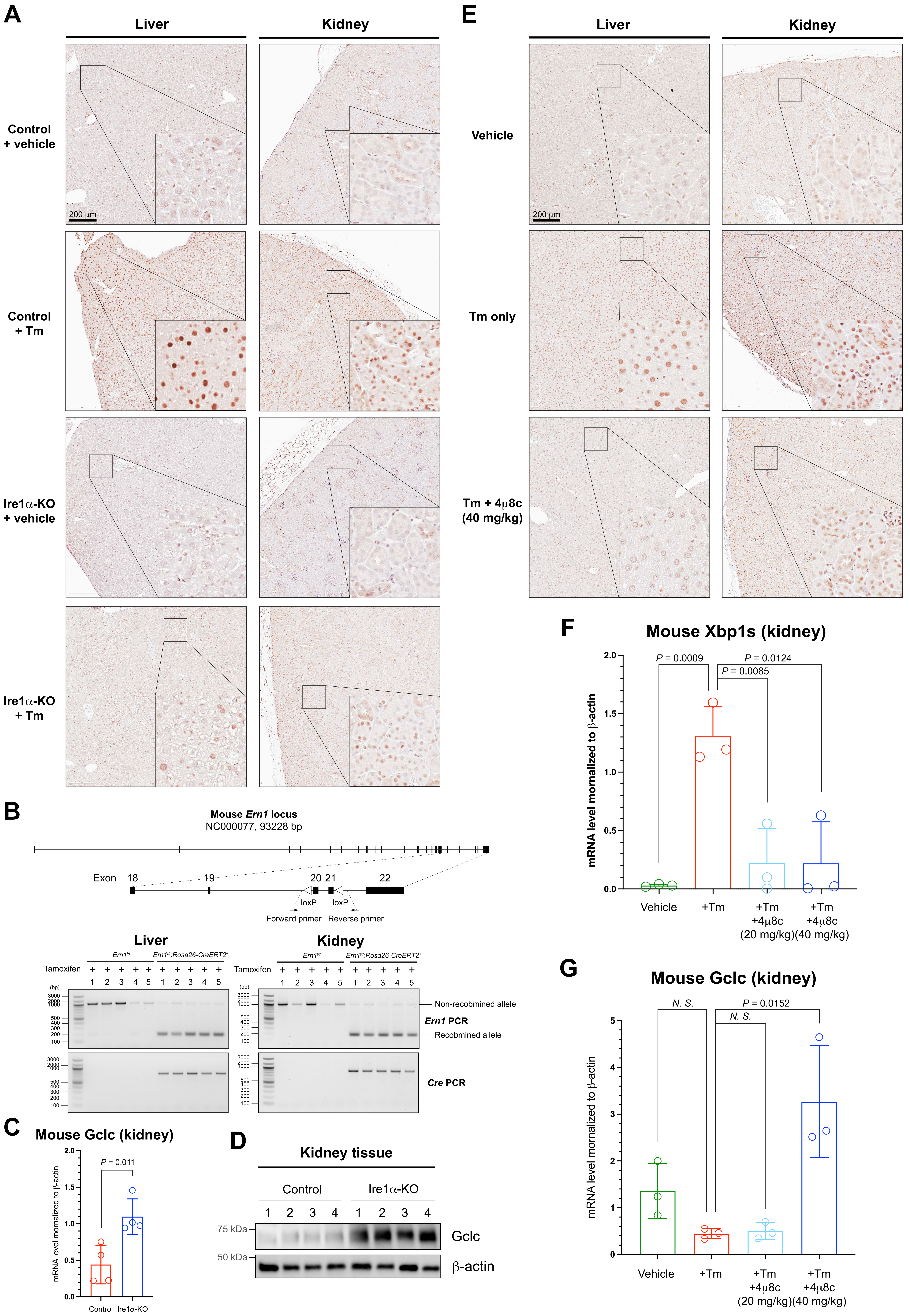

Supplementary Figure 8. **Ire1α deficiency in conditional knockout (KO) mouse tissues. A.** Representative images of immunohistochemistry analysis of spliced Xbp1 (Xbp1s) in liver (left panel) and kidney (right panel) tissues from control (*Ern1*$^{f/f}$ + tamoxifen) or Ire1α-KO (*Ern1*$^{f/f}$*;Rosa26-CreERT2*$^{+}$ + tamoxifen, mouse *Ern1* gene encodes the Ire1α protein) mice treated with either vehicle or tunicamycin (Tm, 10 mg/kg, 6 h) to induce endoplasmic reticulum stress and Xbp1s upregulation. Selected representative regions are enlarged and displayed at the corner; scale bars: 200 μm. Positive Xbp1s signal is represented by strong nuclear staining. **B.** Top: Schematic view of the *Ern1*$^{f/f}$ conditional KO allele. Exons are represented by solid black boxes separated by introns as solid lines. Exon #s are labeled on top of the respective exons. LoxP sites are represented by open arrowhead. PCR primers flanking the loxP sites are represented by black arrows. Bottom: Agarose gel images of *Ern1* and *Cre* genotyping PCR using DNA from liver (left) or kidney (right) of representative control or Ire1α-KO mice. **C.** Quantitative real-time PCR analysis of mouse Gclc mRNA expression level normalized to β-actin in control and Ire1α-KO kidney tissues. Data are presented as mean ± s.d., n = 4. The P value was calculated from unpaired two-tailed Student's t test. **D.** Western blotting analysis of mouse Gclc protein expression level in control and Ire1α-KO kidney tissues. β-actin was used as a loading control. **E.** Representative images of immunohistochemistry analysis of mouse Xbp1s in liver (left panel) and kidney (right panel) tissues from mice treated with vehicle, Tm, or Tm with 40 mg/kg 4μ8c. Selected representative regions are enlarged and displayed at the corner; scale bars: 200 μm. **F** and **G.** Quantitative real-time PCR analysis of (**F**) mouse Xbp1s and (**G**) mouse Gclc mRNA expression levels normalized to β-actin in kidney tissues of mice treated with vehicle, Tm, or Tm with 20 mg/kg or 40 mg/kg 4μ8c. Data are presented as mean ± s.d., n = 3 independent kidneys. P values were calculated from unpaired two-tailed Student's t test. N.S.: not significant. Source data are provided as a Source Data file.

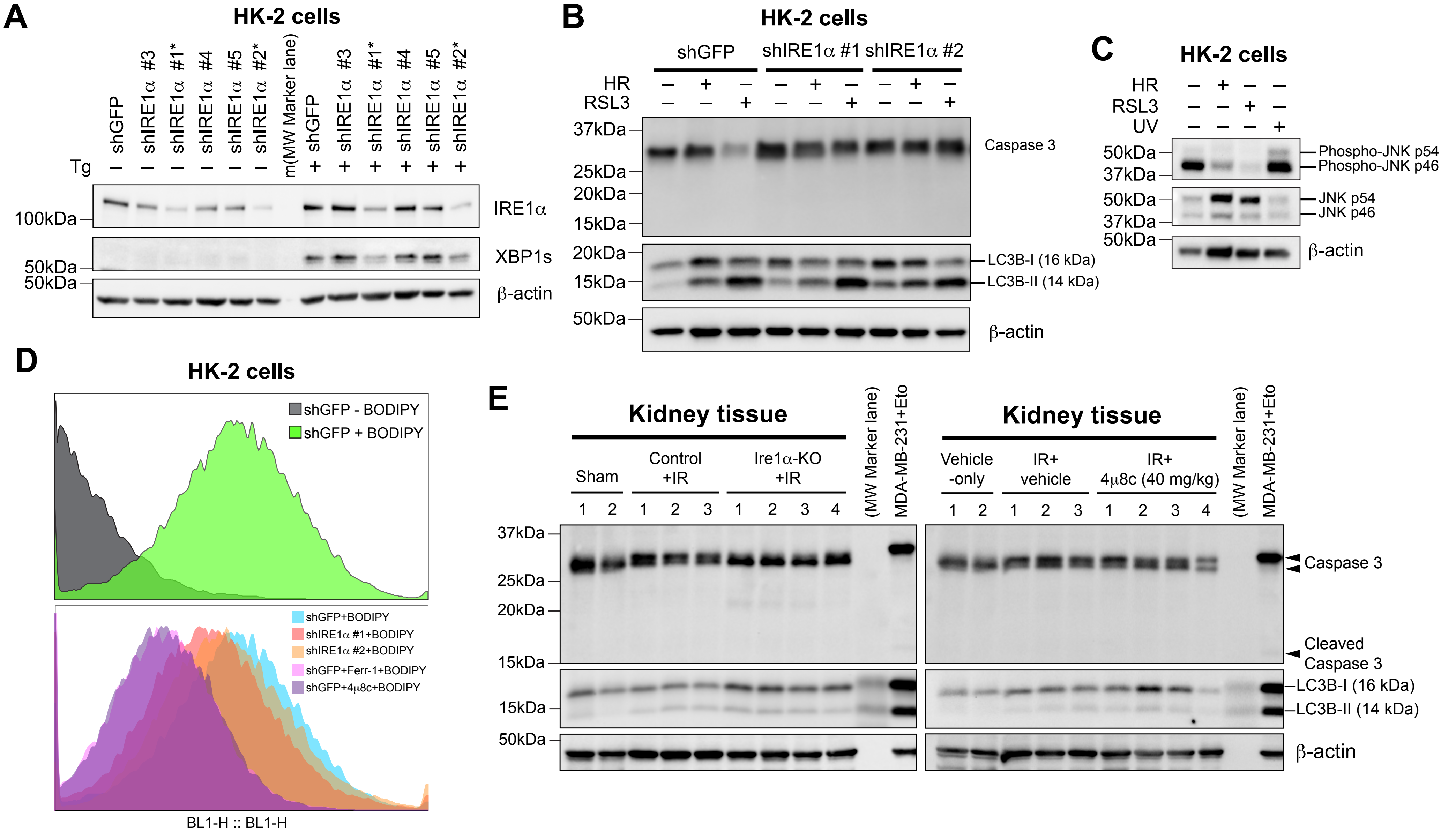

Supplementary Figure 9. A. Western blotting analysis of IRE1α and XBP1s protein expression levels in HK-2 immortalized human kidney proximal tubule epithelial cells transduced with shRNA targeting GFP (shGFP) or IRE1α (shIRE1α) with or without thapsigargin (Tg) treatment. 5 independent shIRE1α were used. shIRE1α with the highest knockdown efficiency (#1 and #2) were marked with *. β-actin was used as a loading control. **B.** Western blotting analysis of apoptosis marker caspase 3 (uncleaved and cleaved), autophagy marker LC3B-I/II levels in HK-2 cells with control (shGFP) or IRE1α (shIRE1α) knockdown treated with hypoxia-reperfusion (HR, 1% O_2 for 16 hours followed by reoxygenation for 2 hours) or RSL3 (1 μM, 3 h). β-actin was used as a loading control. **C.** Western blotting analysis of phospho-/total-JNK in HK-2 cells treated with HR, RSL3 or UV (40 mJ/cm^2, 30 min). β-actin was used as a loading control. **D.** Top panel: the green fluorescence channel distribution of C11-BODIPY staining of control HK-2 cells (+shGFP) when compared to no C11-BODIPY staining. Bottom panel: HK-2 cells with control (+shGFP) or IRE1α (+shIRE1α) knockdown were analyzed with C11-BODIPY 581/591 staining and flow cytometry to measure lipid peroxidation. HK-2 + shGFP cells were also treated with ferropstatin-1 (Ferr-1, 5 μM, 24 h) or 4μ8c (30 μM, 24 h) before C11-BODIPY staining. **E.** Western blotting analysis of apoptosis marker caspase 3 (uncleaved and cleaved) and autophagy marker LC3B-I/II in kidney tissues from sham, control + ischemia-reperfusion (IR), Ire1α-KO + IR mice (left panel) and wild-type mice treated with vehicle, IR + vehicle, or IR + 40 mg/kg 4μ8c. β-actin was used as a loading control. MDA-MB-231 cells treated with 50 μM etoposide (Eto) for 6h were used as a positive control for apoptosis. Source data are provided as a Source Data file.

Supplementary Table 1. sgRNA and shRNA sequences

1. sgRNA sequences used to generate IRE1α KO cells

IRE1α-KO #1 (2 sgRNAs were combined):

TCACCGCCTCGCTGTCGTCG (PAM sequence: CGG, for exon 1)

TCTTGCTTCCAAGCGTATAC (PAM sequence AGG, for exon 4)

IRE1α-KO #2 (Exon 2):

TTGTTTGTGTCAACGCTGGA (PAM sequence: TGG)

IRE1α-KO #3 (Exon 6):

TATGTTATTGACCTCCTGAC (PAM sequence: CGG)

2. shRNA clones (MISSION® shRNA CLONES, Sigma Aldrich)

shIRE1α:

#1: TRCN0000000530. Target Sequence: GAGAAGATGATTGCGATGGAT

#2: TRCN0000000528. Target Sequence: CTACTGGATAAACTTGCTTCA

#3: TRCN0000000529. Target Sequence: CCCATCAACCTCTCTTCTGTA

#4: TRCN0000235531. Target Sequence: CTCAATCAAATGGACTTTAAA

#5: TRCN0000000532. Target Sequence: GAAATACTCTACCAGCCTCTA

shATF4:

#1: TRCN0000013573. Target Sequence: GCCTAGGTCTCTTAGATGATT

#2: TRCN0000013575. Target Sequence: GCCAAGCACTTCAAACCTCAT

shATF6:

#1: TRCN0000017853. Target Sequence: CCCAGAAGTTATCAAGACTTT

#2: TRCN0000017855. Target Sequence: GCAGCAACCAATTATCAGTTT

shGCLC:

#1: TRCN0000344861. Target Sequence: TGAAAGTGCTTCAAGGGTAAT

#2: TRCN0000344862. Target Sequence: CTTCTAAGCCGGATCATATTT

shGFP:

Target Sequence: GCAAGCTGACCCTGAAGTTCAT

3. Construction of pLKO-Tet-On lentiviral shRNA plasmids

shXBP1:

#1: TRCN0000019804

Oligos to be annealed and ligated into pLKO-Tet-On vector through AgeI and EcoRI sites:

Top: 5'**CCGG**GCCTGTCTGTACTTCATTCAA**CTCGAG**TTGAATGAAGTACAGACAGGC**TTTTT**'3

Bottom: 5'**AATTAAAAA**GCCTGTCTGTACTTCATTCAA**CTCGAG**TTGAATGAAGTACAGACAGGC'3

#2: TRCN0000019806

Oligos to be annealed and ligated into pLKO-Tet-On vector through AgeI and EcoRI sites:

Top: 5'**CCGG**GAACAGCAAGTGGTAGATTTA**CTCGAG**TAAATCTACCACTTGCTGTTC**TTTTT**'3

Bottom: 5'**AATTAAAAA**GAACAGCAAGTGGTAGATTTA**CTCGAG**TAAATCTACCACTTGCTGTTC'3

shGFP: SHC314

Oligos to be annealed and ligated into pLKO-Tet-On vector through AgeI and EcoRI sites:

Top: 5'**CCGG**CGTGATCTTCACCGACAAGAT**CTCGAG**ATCTTGTCGGTGAAGATCACG**TTTTT**'3

Bottom: 5' **AATTAAAAA**CGTGATCTTCACCGACAAGAT**CTCGAG**ATCTTGTCGGTGAAGATCACG'3

Supplementary Table 2. List of plasmids and oligo sequences used for *in vitro* transcription

Plasmids (commercial):

Gene	Vector	Vendor & Catalog #	GenBank ID	Enzyme used to linearize
Human *GCLC* (full-length)	pCMV-SPORT6	Horizon Discovery, MHS6278-202759380	BC039894.1	XhoI

Plasmids (constructed, using 5' BamHI-3' XhoI fragment inserted into pGEM-T with 5' T7 promoter):

pGEM-T-hGCLC (fragment #1):

Forward primer: GCACGGATCCAGTTACATGATTGAAGGGACACCAG

Reverse primer: GCACCTCGAGAGCCAGTTCATGATGACTTTAGATATGTTAT

pGEM-T-hGCLC (fragment #2):

Forward primer: GCACGGATCCCAAGACAGTGTCATAACTGATGAAATGA

Reverse primer: GCACCTCGAGACAAGCAAATTGCAAACGAAAAC

pGEM-T-hGCLC (fragment #3):

Forward primer: GCACGGATCCCAAGACAGTGTCATAACTGATGAAATGA

Reverse primer: GCACCTCGAGAGCCAGTTCATGATGACTTTAGATATGTTAT

pGEM-T-hGCLC (fragment #4):

Forward primer: GCACGGATCCTGTACATTTTTAAATTCTTACTCTGGAGCA

Reverse primer: GCACCTCGAGACTTAATTCCTGCATCAGCAAATTTAA

pGEM-T-hGCLC (fragment #5):

Forward primer: GCACGGATCCTTAAACACAACTCTATAGAAAGAAAGGAGATTATTAC

Reverse primer: GCACCTCGAGACAAGCAAATTGCAAACGAAAAC

pGEM-T-gcs-1 (full-length):

Forward primer: GCACGGATCCAATGGGTCTTTTGACGAAAGGTAGT

Reverse primer: GCACCTCGAGCTAATGTGCACGTTTTTGGCTG

pGEM-T-gcs-1 (fragment #1):

Forward primer: GCACGGATCCAATGGGTCTTTTGACGAAAGGTAGT

Reverse primer: GCACCTCGAGCTGCTTTTCTCATCGTCCTGTTC

pGEM-T-gcs-1 (fragment #2):

Forward primer: GCACGGATCCTGAACACTTTGAAACAATTCAATCATC

Reverse primer: GCACCTCGAGCTAATGTGCACGTTTTTGGCTG

Plasmids (constructed, using 5' SacII-3' SacI fragment inserted into pGEM-T with 5' T7 promoter):

pGEM-T-hSLC7A11 (full-length):

Forward primer: GCACCCGCGGGGTTTGTAATGATAGGGCGGC

Reverse primer: GCACGAGCTCCAATAGCTGAGATTACCAGCATGTG

Single-stranded DNA oligonucleotides with 5' T7 promoter:

Human GCLC IRE1α cleavage site:

Wild-type:

Sense:

5'ATTGTAATACGACTCACTATAgggTGATGTTTCTTTTGCAATTGGAATAATCA3'

Anti-sense:

5'TGATTATTCCAATTGCAAAAGAAACATCAcccTATAGTGAGTCGTATTACAAT3'

Mutant:

Sense

5'ATTGTAATACGACTCACTATAgggTGATGTTTCTTTTCCAATTGGAATAATCA3'

Anti-sense:

5'TGATTATTCCAATTGGAAAAGAAACATCAcccTATAGTGAGTCGTATTACAAT3'

C. elegans gcs-1 IRE1α cleavage site:

Wild-type:

Sense:

5’ATTGTAATACGACTCACTATAgggTCGAGGGAACCCCCGGAATGCCTTACGGAGGTCTCATCGC3’

Anti-sense:

5’GCGATGAGACCTCCGTAAGGCATTCCGGGGGTTCCCTCGAcccTATAGTGAGTCGTATTACAAT3’

Mutant:

Sense:

5’ATTGTAATACGACTCACTATAgggTCGAGGGAACCCCCGGAATCCCTTACGGAGGTCTCATCGC3’

Anti-sense:

5’GCGATGAGACCTCCGTAAGGGATTCCGGGGGTTCCCTCGAcccTATAGTGAGTCGTATTACAAT3’

Human SLC7A11 IRE1α cleavage site:

Site 1:

Wild-type:

Sense:

5’GTAATACGACTCACTATAggTCTCCAAAGGAGGTTACCTGCAGGGAAATGTTAACGGGAG3’

Anti-sense:

5’CTCCCGTTAACATTTCCCTGCAGGTAACCTCCTTTGGAGAccTATAGTGAGTCGTATTAC3’

Mutant:

Sense

5’GTAATACGACTCACTATAggTCTCCAAAGGAGGTTACCTCCAGGGAAATGTTAACGGGAG3’

Anti-sense:

5’CTCCCGTTAACATTTCCCTGGAGGTAACCTCCTTTGGAGAccTATAGTGAGTCGTATTAC3’

Site 2:

Wild-type:

Sense:

5’GTAATACGACTCACTATAggTATCACACTGGGAGGCAATGCAATGTGGTTACCTGGTCCT3’

Anti-sense:

5'AGGACCAGGTAACCACATTGCATTGCCTCCCAGTGTGATAccTATAGTGAGTCGTATTAC3'

Mutant:

Sense

5'GTAATACGACTCACTATAggTATCACACTGGGAGGCAATCCAATGTGGTTACCTGGTCCT3'

Anti-sense:

5'AGGACCAGGTAACCACATTGGATTGCCTCCCAGTGTGATAccTATAGTGAGTCGTATTAC3'

Site 3:

Wild-type:

Sense:

5'GTAATACGACTCACTATAggTAAGGCTGTATCATTTAATGCTATGAGATACATTGTTTTC3'

Anti-sense:

5'GAAAACAATGTATCTCATAGCATTAAATGATACAGCCTTAccTATAGTGAGTCGTATTAC3'

Mutant:

Sense

5'GTAATACGACTCACTATAggTAAGGCTGTATCATTTAATCCTATGAGATACATTGTTTTC3'

Anti-sense:

5'GAAAACAATGTATCTCATAGGATTAAATGATACAGCCTTAccTATAGTGAGTCGTATTAC3'

Supplementary Table 3. List of qPCR primers

Human *SLC7A11*:

Forward: 5’ GCCCAGATATGCATCGTCCTT 3’

Reverse: 5’ ATGACGAAGCCAATCCCTGTA 3’

Human *GCLC*:

Forward: 5’ CTGTTGCAGGAAGGCATTGA 3’

Reverse: 5’ AACAGTGTCAGTGGGTCTCTAATAAAGA 3’

Human *ACTB*:

Forward: 5’ ATCCGCCGCCCGTCCACA 3’

Reverse: 5’ ACCATCACGCCCTGGTGCCT 3’

Mouse *Gclc*:

Forward: 5’ CTGTCTGACCCCTGTGCTGAT 3’

Reverse: 5’ GCAAACTAGAGAAGGGCAGGAA 3’

Mouse *Actb*:

Forward: 5’-TCCTAGCACCATGAAGATCAAGATC-3’

Reverse: 5’-CTGCTTGCTGATCCACATCTG-3’
