# Supplementary material for: IRE1α determines ferroptosis sensitivity through regulation of glutathione synthesis
Source: Nat Commun. 2024 May 15;15:4114. doi: 10.1038/s41467-024-48330-0 (PMC11096184; doi:10.1038/s41467-024-48330-0)
Supplement: Supplementary file 3 — Reporting Summary [file 41467_2024_48330_MOESM3_ESM.pdf]

nature portfolio

Corresponding author(s): Dadi Jiang and Albert C. Koong

Last updated by author(s): Apr 4, 2024

Reporting Summary

Nature Portfolio wishes to improve the reproducibility of the work that we publish. This form provides structure for consistency and transparency in reporting. For further information on Nature Portfolio policies, see our Editorial Policies and the Editorial Policy Checklist.

Statistics

For all statistical analyses, confirm that the following items are present in the figure legend, table legend, main text, or Methods section.

n/a	Confirmed	
☐	☒	The exact sample size (*n*) for each experimental group/condition, given as a discrete number and unit of measurement
☐	☒	A statement on whether measurements were taken from distinct samples or whether the same sample was measured repeatedly
☐	☒	The statistical test(s) used AND whether they are one- or two-sided<br>*Only common tests should be described solely by name; describe more complex techniques in the Methods section.*
☒	☐	A description of all covariates tested
☒	☐	A description of any assumptions or corrections, such as tests of normality and adjustment for multiple comparisons
☐	☒	A full description of the statistical parameters including central tendency (e.g. means) or other basic estimates (e.g. regression coefficient) AND variation (e.g. standard deviation) or associated estimates of uncertainty (e.g. confidence intervals)
☐	☒	For null hypothesis testing, the test statistic (e.g. F, t, r) with confidence intervals, effect sizes, degrees of freedom and P value noted<br>*Give P values as exact values whenever suitable.*
☒	☐	For Bayesian analysis, information on the choice of priors and Markov chain Monte Carlo settings
☒	☐	For hierarchical and complex designs, identification of the appropriate level for tests and full reporting of outcomes
☐	☒	Estimates of effect sizes (e.g. Cohen's *d*, Pearson's *r*), indicating how they were calculated

Our web collection on statistics for biologists contains articles on many of the points above.

Software and code

Policy information about availability of computer code

Data collection	An Attune NxT flow cytometer (Thermo Fisher Scientific) was used to collect flow cytometry data. IncuCyte S3 (Sartorius) running software version 2019B Rev2 was used to collect live cell imaging data. Cytation 5 (BioTek) running Gen5 3.13 software was used to collect absorbance and luminescence readings from colorimetric and luminescence assays, respectively. QuantStudio 5 Real-Time PCR System (Applied Biosystems) running software v1.5.1 was used to collect quantitative real-time PCR data. Tri-Carb® Liquid Scintillation Analyzer (PerkinElmer, Model 4810TR) was used to collect radioactivity data for cystine uptake assay. Image Lab V4.0 (Bio-Rad Laboratories) was used to collect gel images.
Data analysis	Microsoft Excel (Version 16.80 for Mac) and GraphPad Prism 9.0 were used for bar graph output and statistic analysis. FlowJo (V10) was used for flow cytometry data analysis.

For manuscripts utilizing custom algorithms or software that are central to the research but not yet described in published literature, software must be made available to editors and reviewers. We strongly encourage code deposition in a community repository (e.g. GitHub). See the Nature Portfolio guidelines for submitting code & software for further information.

Data

Policy information about availability of data

All manuscripts must include a data availability statement. This statement should provide the following information, where applicable:

- Accession codes, unique identifiers, or web links for publicly available datasets
- A description of any restrictions on data availability
- For clinical datasets or third party data, please ensure that the statement adheres to our policy

The Cancer Therapeutics Response Portal (CTRP, V2.1) is a web-based publicly accessible database (https://portals.broadinstitute.org/ctrp.v2.1/). The data generated in this study are provided in the Supplementary Information/Source Data file. Source data are provided with this paper.

Research involving human participants, their data, or biological material

Policy information about studies with human participants or human data. See also policy information about sex, gender (identity/presentation), and sexual orientation and race, ethnicity and racism.

Reporting on sex and gender	N/A
Reporting on race, ethnicity, or other socially relevant groupings	N/A
Population characteristics	N/A
Recruitment	N/A
Ethics oversight	N/A

Note that full information on the approval of the study protocol must also be provided in the manuscript.

Field-specific reporting

Please select the one below that is the best fit for your research. If you are not sure, read the appropriate sections before making your selection.

☒ Life sciences ☐ Behavioural & social sciences ☐ Ecological, evolutionary & environmental sciences

For a reference copy of the document with all sections, see nature.com/documents/nr-reporting-summary-flat.pdf

Life sciences study design

All studies must disclose on these points even when the disclosure is negative.

Sample size	The sample size for each experiment is described in figures or figure legends. The sample sizes were not predetermined by statistical tools. All the experiments were performed using sample sizes based on previous experience in the lab. We made an effort to avoid unnecessary use of animals.
Data exclusions	No animals were excluded from in vivo studies.
Replication	Multiple independent repeats were included for related experiments. Each experiment was performed for at least twice with successful replication to make sure similar results are reproducible.
Randomization	For the C. elegans survival studies, each strain of worms were randomly assigned to -DGLA or +DGLA group. For the mouse kidney IRI studies with 4u8c and liproxstatin-1 treatment, 10-12 weeks old C57BL/6 mice were randomly assigned to the different treatment groups. For the mouse kidney IRI studies using wild-type or Ire1a-null mice as well as cell-based experiments no randomization was applied because these were defined groups, e.g. non-targeting vs. IRE1a-KO.
Blinding	For cell-based experiments, Western blotting and flow cytometry analysis, blinding was not possible because the experiments were performed by individual researchers. For histological evaluation and immunohistochemical analysis of mouse kidney tissue, when quantifying damaged or 4-HNE+ tubules, the stained slides were quantified in a blinded way.

Reporting for specific materials, systems and methods

We require information from authors about some types of materials, experimental systems and methods used in many studies. Here, indicate whether each material, system or method listed is relevant to your study. If you are not sure if a list item applies to your research, read the appropriate section before selecting a response.

Materials & experimental systems

n/a	Involved in the study
☐	☒ Antibodies
☐	☒ Eukaryotic cell lines
☒	☐ Palaeontology and archaeology
☐	☒ Animals and other organisms
☒	☐ Clinical data
☒	☐ Dual use research of concern
☒	☐ Plants

Methods

n/a	Involved in the study
☒	☐ ChIP-seq
☐	☒ Flow cytometry
☒	☐ MRI-based neuroimaging

Antibodies

Antibodies used

Primary antibodies (1:1000 dilution in 3% BSA): IRE1alpha (3294, Cell Signaling), GCLC (sc-390811, Santa Cruz Biotechnology), GCLM (sc-22754, Santa Cruz Biotechnology), GPX4 (MAB5457, R&D Systems), SLC7A11 (12691, Cell Signaling), ACSL4 (SAB2701949, Sigma Aldrich), XBP1 (ab220783, Abcam), luciferase (NB600-307, Novus Biologicals), calreticulin (ab2907, Abcam), beta-actin (sc-1615, Santa Cruz Biotechnology), 4-HNE (ab46545, Abcam), PERK (3192, Cell Signaling), phospho-GCN2 (ab75836, Abcam), GCN2 (3302, Cell Signaling), phospho-eIF2alpha (9721, Cell Signaling), eIF2alpha (2103, Cell Signaling), ATF4 (11815, Cell Signaling), ATF6 (CosmoBio, BAM-73-500-EX), phospho-JNK (9251, Cell Signaling), JNK (9252, Cell Signaling), LC3B (NB600-1384, Novus), Caspase-3 (9662, Cell Signaling), HSP90 (sc-13119, Santa Cruz Biotechnology), and GAPDH (8884, Cell Signaling). HRP-conjugated secondary antibodies: goat anti-rabbit IgG (111-035-144, Jackson ImmunoResearch), goat anti-mouse IgG (115-035-003, Jackson ImmunoResearch) at 1:3000 dilution in 3% BSA.

Validation

All antibodies used in our study have been validated and detailed information could be found on the manufacture websites as listed below. Some of them have also been validated in our study using either knockout or overexpression strategies.
IRE1alpha: https://www.cellsignal.com/products/primary-antibodies/ire1a-14c10-rabbit-mab/3294
GCLC: https://www.scbt.com/p/gamma-gcsc-antibody-h-5
GCLM: https://www.scbt.com/p/gamma-gcsm-antibody-fl-274
GPX4: https://www.rndsystems.com/products/human-mouse-rat-glutathione-peroxidase-4-gpx4-antibody-565320_mab5457
SLC7A11: https://www.cellsignal.com/products/primary-antibodies/xct-slc7a11-d2m7a-rabbit-mab/12691
ACSL4: https://www.sigmaaldrich.com/US/en/product/sigma/sab2701949
XBP1: https://www.abcam.com/xbp1-antibody-epr22004-ab220783.html
Luciferase: https://www.novusbio.com/products/luciferase-antibody-luci-21-1-107_nb600-307
Calreticulin: https://www.abcam.com/calreticulin-antibody-er-marker-ab2907.html
Beta-actin: https://www.scbt.com/p/actin-antibody-c-11
4-HNE: https://www.4adi.com/4adi/anti-4-hydroxynonenal-hne-antiserum-11750-p.html
PERK: https://www.cellsignal.com/products/primary-antibodies/perk-c33e10-rabbit-mab/3192?_requestid=4539502
phospho-GCN2: https://www.abcam.com/products/primary-antibodies/gcn2-phospho-t899-antibody-epr2320y-ab75836.html
GCN2: https://www.cellsignal.com/products/primary-antibodies/gcn2-antibody/3302
phospho-eIF2alpha: https://www.cellsignal.com/products/primary-antibodies/phospho-eif2a-ser51-antibody/9721
eIF2alhpa: https://www.cellsignal.com/products/primary-antibodies/eif2a-l57a5-mouse-mab/2103
ATF4: https://www.cellsignal.com/products/primary-antibodies/atf-4-d4b8-rabbit-mab/11815
ATF6: https://www.cosmobiousa.com/products/anti-atf6-alpha-mab-clone-1-7
phospho-JNK: https://www.cellsignal.com/products/primary-antibodies/phospho-sapk-jnk-thr183-tyr185-antibody/9251
JNK: https://www.cellsignal.com/products/primary-antibodies/sapk-jnk-antibody/9252
LC3B: https://www.novusbio.com/products/lc3b-antibody_nb600-1384
Caspase-3: https://www.cellsignal.com/products/primary-antibodies/caspase-3-antibody/9662
HSP90: https://www.scbt.com/p/hsp-90alpha-beta-antibody-f-8?gclid=CjwKCAjwyNSoBhA9EiwA5aYlbzK8WDrG7thZKLk_YpIDdVl2hH4O2MxGtkZ4euQFsDNy_gp0_Ftg5hoC5gUQAvD_BwE
GAPDH: https://www.cellsignal.com/products/antibody-conjugates/gapdh-d16h11-xp-rabbit-mab-hrp-conjugate/8884
HRP-conjugated goat anti-rabbit IgG: https://www.jacksonimmuno.com/catalog/products/111-035-144
HRP-conjugated goat anti-mouse IgG: https://www.jacksonimmuno.com/catalog/products/115-035-003

Eukaryotic cell lines

Policy information about cell lines and Sex and Gender in Research

Cell line source(s)

The MDA-MB-231 and HK-2 cell lines were purchased from the American Type Culture Collection. The Panc-1 cell line was purchased from Sigma Aldrich. The Ire1+/+ and Ire1-/- MEFs were obtained from Dr. Fumihiko Urano at Washington University in St. Louis.

Authentication

The MDA-MB-231 cell line was authenticated by Short Tandem Repeat (STR) DNA Fingerprinting at the MD Anderson Cytogenetics and Cell Authentication Core (CCAC). STR DNA profiling, also known as DNA fingerprinting, offers the greatest value for cell line authentication. The assay is based on screening regions of microsatellite instability with defined tri- or tetrad-nucleotide repeats located throughout the chromosomes. PCR reactions using primers on non-repetitive flanking those regions will generate PCR products of different sizes based on the number of repeats in the region; the size of these PCR products is determined by capillary electrophoresis. By combining between 8 and 16 STR loci, such as D5S818, D13S317, D7S820, D16S539, vWA, TH01, TPOX, and CSF1PO, it is possible to uniquely identify a sample. The CCAC assay screens 16 loci using the Promega Powerplex 16 HS kit. The assay also includes matching the STR profiles against an internal database

	comprised of public profiles and profiles that are unique to cell lines developed by MDACC investigators. Our current database has over 4000 profiles. The other cell lines were not authenticated.
Mycoplasma contamination	All cell lines were free of mycoplasma at the time of the assay tested with the MycoAlert Mycoplasma Detection Kit (Lonza).
Commonly misidentified lines (See ICLAC register)	No cell line used in this study has been found in the International Cell Line Authentication Committee (ICLAC) database of commonly misidentified cell lines (version 11).

Animals and other research organisms

Policy information about studies involving animals; ARRIVE guidelines recommended for reporting animal research, and Sex and Gender in Research

Laboratory animals	10-12 weeks old C57BL6 mice, Ern1f/f and Rosa26-CreERT2 mice were produced or maintained through breeding at the MD Anderson Small Animal Facility. Rodent housing conditions used in this study are: temperature set point: 72F; high limit: 74F; low limit: 70F. Humidity set point: 45%; high limit: 55%; low limit: 40%. Light cycle: 12 hour light/dark. The mice had ad libitum access to water and food. All the C. elegans strains used in this study, including N2 [var Bristol] (wild-typ), RB925 [ire-1(ok799) II] (ire-1 deletion), and SJ30 [ire-1(zc14) II; zcIs4 V] (ire-1 kinase domain mutant) were obtained from the Caenorhabditis Genetics Center (CGC) at the University of Minnesota. All strains were cultured and maintained at 20 °C on solid nematode growth medium (NGM) seeded with E. coli OP50 as a standard diet. For the lifespan assay, age-synchronized worms were obtained by hatching the eggs from gravid worms in 6 cm NGM petri plates and incubating at 20°C until reaching the L4 stage.
Wild animals	No wild animals were involved in this study.
Reporting on sex	Sex was not considered in the study design.
Field-collected samples	No field-collected samples were involved in this study.
Ethics oversight	This research complies with all relevant ethical regulations of the University of Texas MD Anderson Cancer Center. All animal studies were performed in accordance with a protocol approved by the Institutional Animal Care and Use Committee (IACUC protocol # 00001769).

Note that full information on the approval of the study protocol must also be provided in the manuscript.

Flow Cytometry

Plots

Confirm that:

- [x] The axis labels state the marker and fluorochrome used (e.g. CD4-FITC).
- [x] The axis scales are clearly visible. Include numbers along axes only for bottom left plot of group (a 'group' is an analysis of identical markers).
- [ ] All plots are contour plots with outliers or pseudocolor plots.
- [x] A numerical value for number of cells or percentage (with statistics) is provided.

Methodology

Sample preparation	Cells were plated at a density of 3 x 10^5 cells in 2 ml medium per well in 6-well plates (Corning Falcon). The next day, the medium in each well was replaced with 2 ml fresh medium with DMSO or 10 uM erastin. 10 hours later, the cells in each well were washed with PBS once, then incubated with 1 ml PBS containing 2 uM C11-BODIPY 581/591 (Invitrogen) at 37 °C for 15 min. After a brief PBS rinse, the cells were trypsinized and resuspended in 500 ml PBS before flow cytometry analysis.
Instrument	Attune NxT flow cytometer (Thermo Fisher Scientific)
Software	Using Attune software to collect data and FlowJo_V10 software to analyze data.
Cell population abundance	A minimum of 10,000 single cells were analyzed per sample.
Gating strategy	Initial cell population gating (SSC-Area vs FSC-Area) was used to select the live cell population. Then the live cell population was separated on an FSC-Area vs FSC-Height plot to exclude cell doublets or aggregates and only single cells were used for subsequent analyses. Then the live/single cell population was plotted using the BL1-Height channel as a histogram. The final plots were presented as Multigraph Overlay to facilitate comparison between samples. All the original plots as well as a figure exemplifying the gating strategy would be provided upon request.

- [ ] Tick this box to confirm that a figure exemplifying the gating strategy is provided in the Supplementary Information.
